# Supplementary material for: What Works for Whom? The Influence of Problem Severity, Maladaptive Perfectionism, and Perceived Parental Pressure on the Effectiveness of a School-Based Performance Anxiety Program
Source: Behav Sci (Basel). 2025 Mar 28;15(4):436. doi: 10.3390/bs15040436 (PMC12023957; doi:10.3390/bs15040436)
Supplement: Supplementary file 1 [file behavsci-15-00436-s001.zip › behavsci-3418847-supplementary.pdf]

Supplementary Materials

Supplementary Table S1. Means, standard deviations and group differences between adolescents who attended less than four sessions (*N* = 49) and adolescents who attended four or more sessions (*N* = 46).

	Less than four sessions (*N* = 49)	Four or more sessions (*N* = 46)	Differences between groups
	M (*SD*)	*M* (*SD*)	*t* (*p*)
Test anxiety (T1)	1.81 (0.77)	2.30 (0.61)	**-4.44 (< .001)**
Fear of failure (T1)	1.85 (1.00)	2.57 (0.98)	**-3.93 (< .001)**
Self-oriented perfectionism-criticism (T1)	2.09 (0.85)	2.86 (0.94)	**-4.19 (< .001)**
Socially prescribed perfectionism (T1)	2.29 (0.93)	2.83 (0.82)	**-2.95 (.00)**
Perceived parental pressure (T2)	2.17 (1.15)	2.32 (1.11)	-0.64 (.52)

Note. Significant results are in bold.
